# Lysins – a new armamentarium for the treatment of bone and joint infections?

**DOI:** 10.5194/jbji-7-187-2022

**Published:** 2022-09-06

**Authors:** Parham Sendi, Tristan Ferry

**Affiliations:** 1 Institute for Infectious Diseases, University of Bern, Bern, Switzerland; 2 Hospices Civils de Lyon, 69004 Lyon, France; 3 Université Claude Bernard Lyon 1, 69100 Villeurbanne, France; 4 Centre de Références des IOA Complexes de Lyon, CRIOAc Lyon, 69004 Lyon, France; 5 StaPath Team, Centre International de Recherche en Infectiologie, CIRI, Inserm U1111, CNRS UMR5308, ENS de Lyon, UCBL1, 69008 Lyon, France; ℹ JBJI Editor-in-chief

The treatment of bone and joint infections (BJIs) is complex. It requires a well-functioning and frequently interacting multi-disciplinary team with expertise in orthopedic and trauma surgery, infectious diseases and clinical microbiology, musculoskeletal radiology, and plastic surgery, among others (Vasoo et al., 2019). Moreover, understanding of the biofilm pathogenesis must be incorporated into the treatment concepts (Tande and Patel, 2014). Considering the worrisome increasing trend of BJIs caused by multi-drug-resistant bacteria (Papadopoulos et al., 2019), additional challenges have entered the field, both from a treatment and infection control perspective.

While traditional antibiotics are losing their edge, new compounds, small molecules, and biological and chemical structures are potentially new weapons. Phage therapy is one of them. Phages can be injected intravenously or locally. Frankly, phage therapy is not a new concept. Fortunately, eastern European countries (e.g., Georgia) have kept phage therapy alive since 1923 (Dublanchet and Bourne, 2007). Ironically, one could say that, as we are facing the postantibiotic era, we are looking for recipes from the preantibiotic era. The usefulness of these compounds must be carefully reviewed, considering the most up-to-date principles of research.

Meanwhile, numerous publications have reported their success in using phage therapy for BJIs and implant-associated infections caused by multi-drug-resistant bacteria (Cano et al., 2021; Clarke et al., 2020; Ferry et al., 2020, 2021; Schoeffel et al., 2022). Of note, only experiences with positive outcomes have been published. There are no randomized clinical trials in the field of BJIs that have been completed.

As with every treatment, there are also drawbacks with phage therapy. Considering their specificity for the infecting organism, phages are not an ideal candidate for mass production. Patenting phages is not beneficial for the same reason and in light of the magnitude of existing organisms on this planet. There are also hurdles when phages are administered locally. Due to the high specificity of phages, the organism causing the infection must be isolated *prior to* surgery. Then, it can be applied during surgery after selecting the matching phage cocktail. If phage therapy is considered *after* the surgery, local treatment with phages is not easily feasible, except in patients with periprosthetic joint infections. In these infections, phages can be injected directly into the joint, but the implant–bone interface cannot be reached. Taken together, local phage therapy is complex and highly specific. It is an unsuitable method for preventing infection.

Lysins, which are biologic enzymes, are a promising alternative to phage therapy. While phages replicate within the targeted bacteria, they must also find their way out of their host bacteria (to infect new targeted bacteria). Phages do this by producing lysins, small molecules that destroy the bacterial cell wall from inside, and thus facilitate the expulsion of hundreds of new virions. Basic science research demonstrated that purified lysins destroy bacterial cell walls, also when added extracellularly (Fischetti, 2008; Fischetti et al., 2006). Hitherto, there have been no reports of bacterial resistance to the lysins, whereas acquisition of phage resistance has been described (Oechslin, 2018). Thus, purified lysins revealed their potential as a direct agent against bacterial infections.

Exebacase (previously named CF-301) and CF-296 are recombinant lysins derived from a *Streptococcus suis* phage. Interestingly, the spectrum of action of these lysins is broader in comparison with a specific *S. aureus* phage. They are also active against coagulase-negative staphylococci (CoNS). The activity against CoNS aligns with the following advantages. (i) CoNS are most frequently involved in implant-associated BJIs. (ii) There are currently 47 species recognized in the genus *Staphylococcus* (Becker et al., 2014). (iii) There are no available phages targeting these bacteria. In the phase-2 trial, exebacase has been used in patients with *S. aureus* bloodstream infections and endocarditis (Fowler et al., 2020) and is currently being investigated in the phase-3 trial for safety and efficacy (NCT04160468).

In the field of BJIs, exebacase has been investigated in vitro for its activity against *S. aureus* and CoNS biofilm (Schuch et al., 2017) and against methicillin-resistant *S. aureus* osteomyelitis in animals (Karau et al., 2019). CF-296 has been investigated for its activity against methicillin-resistant *S. aureus* osteomyelitis in animals (Karau et al., 2021). Currently, the clinical experience of exebacase is limited to a few patients with relapsing *S. epidermidis* periprosthetic joint infections (Karau et al., 2019). Further research is necessary to elucidate both the techniques of administration and local drug delivery of lysins in BJIs. Moreover, their relevance as an adjunctive therapy to antibiotics needs to be explored.

In this issue of *Journal of Bone and Joint Infection*, Karau et al. (2022) investigate the antibacterial activity of locally delivered exebacase or CF-296 in combination with intravenous daptomycin or saline against methicillin-resistant *S. aureus* in an experimental animal model of implant-associated osteomyelitis. The authors differentiated the efficacy results in implant and bone cultures. In implant cultures, exebacase alone or with daptomycin as well as CF-296 with daptomycin were more active than daptomycin alone or CF-296 alone. In bone cultures, CF-296 with daptomycin was more active than either CF-296 alone or daptomycin alone. There was no difference between the two lysins' activity when delivered locally in conjunction with systemic daptomycin, whether based on bone or implant cultures. The results of this study are important to note for the following reasons. First, no emergence of resistance was found to either lysin. Second, the activity of lysins may be different against bone and implant biofilms when administered alone. Third, the combination of systemically administered antibiotics and locally delivered lysins needs to be further explored. Indeed, it would be required to identify the potential best match of a specific antimicrobial agent with a specific lysin to target the causative microorganism in BJIs.

In line with the en vogue term “precision medicine”, lysins are promising options with considerable potential as add-on agents in the treatment armamentarium against bacterial infections, including BJIs. They may turn out to be key local or systemic anti-biofilm agents in the future and could compensate for the current challenges with antibiotic treatment against staphylococcal BJIs, including those related to rifampin. Of note, there are also BJI treatment challenges with fluoroquinolones, including the increasing resistance in Gram-negative bacteria. Lysins against Gram-negative lysins are in the pipeline.

## Data Availability

No data sets were used in this article.
